# The Role of Polypropylene Microfibers in Thermal Properties and Post-Heating Behavior of Cementitious Composites

**DOI:** 10.3390/ma13122676

**Published:** 2020-06-12

**Authors:** Mohammad R. Irshidat, Nasser Al-Nuaimi, Mohamed Rabie

**Affiliations:** Center for Advanced Materials (CAM), Qatar University, P.O. Box 2713, Doha 00000, Qatar; anasser@qu.edu.qa (N.A.-N.); m.rabie@qu.edu.qa (M.R.)

**Keywords:** cementitious composites, polypropylene fibers, thermal, mechanical, fire

## Abstract

This paper experimentally studied the effect of polypropylene (PP) microfibers on thermal and post-heating mechanical behaviors of cementitious composites. Cement mortars with small dosage of polypropylene fibers were prepared, heated at various temperatures (150 °C, 200 °C, 450 °C, and 600 °C), and then tested. The investigated parameters include residual compressive and flexural strengths, elastic modulus, fracture energy, stress intensity factors, failure modes, microstructure (scanning electron microscopy (SEM) imaging), thermal conductivity, heat flow (differential scanning calorimetry (DSC) test), mass loss (thermogravimetric analysis (TGA) test), and chemical composition (XRD analysis). The results showed the efficiency of PP fibers to enhance the post-heating behavior and the residual mechanical properties of cement mortar after heating. The presence of PP fibers did not affect the heat flow and the mass loss of cement mortar at room temperature. However, heating cement mortar at temperature beyond the melting point of the fibers negatively affected its thermal behavior. The presence of PP fibers played a major role in bridging the cracks and mitigating their propagation. Once the melting point of the polypropylene fibers is exceeded, the fibers melted and created extra voids in the microstructure of concrete.

## 1. Introduction

Polypropylene (PP) fibers with their lightweight character, corrosion resistance, and relatively low cost are incorporated in concrete production to enhance its mechanical strengths, cracking patterns, durability, and fire resistance [1,2,3,4,5,6]. In the event of fire, the high temperatures usually causes dehydration of the portlandite and calcium silicate hydrate (C-S-H) gel, which generates water steam thus increase the pore pressure inside the concrete. The generated pore pressure causes splitting of concrete cover or structural spalling [7,8]. Spalling can be considered as one of the major concerns when dealing with concrete structures under fire [8,9,10,11]. One of the proposed methods to control and mitigate concrete spalling during fire is to incorporate polypropylene fibers within the concrete mix [3,5,6,7,12,13,14,15]. Once the melting point of the polypropylene fibers is exceeded, the fibers melt and create extra capillary pores in the microstructure of concrete. The additional pores allow the water steam generated due to the dehydration process to release thus reducing the pore pressure and delaying the spalling. The published literature reflects the fact that the integration of polypropylene fibers in cement-based materials not only enhances its structural performance but also improves its fire resistance [5,6,12,13,14,15]. 

In addition, other studies demonstrate that adding PP fibers improves the residual mechanical properties of cementitious composites after fire [13,16]. Ezziane et al. [13] investigated the effect of PP fibers on the behavior of cement mortar after being exposed to elevated temperatures in the range of 400 °C to 1000 °C. Their results showed that incorporating PP fibers enhanced the mechanical properties of the mortar at room temperature and when heated up to certain temperature. The bending strength and fracture energy were improved in the presences of the fibers after heating up to 500 °C and 400 °C, respectively. Beyond these temperatures, the bending strength and fracture energy of the fiber-reinforced mortar was less than that of plain mortar. Aydin et al. [16] investigated the influence of PP fibers’ incorporation on high temperature behavior of cement mortars exposed to elevated temperatures up to 900 °C. Their results showed an enhancement in the residual mechanical properties of cement mortar in the presence of PP fibers. Eidan et al. [14] reported that PP fiber-reinforced concrete experienced less, shallower, and shorter cracks than plain concrete specimens when exposed to a heating-cooling cycle of 600 °C. They also showed that the amount and the length of the fibers had a significant effect on cracking prevention. Noumowe [5] showed that using PP fibers affected the porosity of the high-strength concrete when exposed to high temperature. When PP fiber-reinforced high strength concrete was heated up to 200 °C, fibers melted creating extra voids and small channels in the concrete microstructure. Szeląg [17] investigated the local microstructure of the PP fiber-reinforced cement paste when exposed to elevated temperature. His scanning electron microscopy (SEM) images confirmed the melting of the fibers after heating which caused extra void in the microstructure. 

Most of the published literature focused on investigating the effect of PP fibers on the structural performance and residual mechanical properties of heat-damaged cementitious materials. Limited research was directed toward investigating the effect of the fibers on the thermal properties, chemical composition degradation and fracture energy of cement-based materials. These properties are important to estimate the ability of the cementitious specimens to survive after being exposed to fire. Accordingly, the current study investigates the effect of PP fibers on the mechanical and thermal properties of cement mortar after exposed to elevated temperatures of 150 °C, 200 °C, 450 °C, and 600 °C. The effect of heating level and mixture design on the residual compressive strength, residual flexural strength, modulus of elasticity, failure pattern, fracture energy, stress intensity factor, and thermal conductivity is studied. The role of the fibers in the post-heating behavior of the mortar is discussed through thermal analysis and microstructure investigation. The thermal behavior of cement mortar with PP fibers is investigated using differential scanning calorimetry (DSC) and thermogravimetric analysis (TGA) tests. The microstructure of the heat-damaged specimens is investigated using SEM imaging.

## 2. Experimental Details

### 2.1. Materials and Mix Proportions 

The materials used in this research included Portland cement, silica sand, Tap water, polypropylene fibers, and superplasticizer. The cement was commercially available Portland cement with chemical composition depicted in Table 1. The silica sand had specific gravity of 2.56, fines modulus of 2.31, water absorption of 1.87%, and conformed with the ASTM C778-17 Standard [18]. Monofilament polypropylene microfibers with 12 mm length, 34-micrometer diameter, 910 kg/m^3^ density, and 160 °C melting point received from Sika^®^ (Doha, Qatar) and used in this study. Commercially available polycarboxylate ether superplasticizer namely “PC 485” provided by EPSILONE (Beirut, Lebanon) was used in all mixes for improved workability and consistency.

Three cement mortar batches were prepared in this study; one control (plain mortar) whereas the other two contains different dosages of PP fibers. 0.1% and 0.05% of PP fibers by weight of cement (equivalent to 0.08% and 0.04% volume proportion of the mortar) were used to prepare mortar batches used to cast compression and flexural specimens, respectively. These dosages represent the optimal amount of PP fibers required to enhance the compression and flexural strengths of cement mortar according to previous work published by the authors [19]. The water to cement (w/c) ratio was fixed at 0.48 for all mixes. Superplasticizer was used by 0.01% (by weigh of cement) to enhance the workability of the mix. The details of mix designs are summarized in Table 2.

### 2.2. Mortar Casting and Specimen Preparation

Cement mortar was casted according to ASTM C305 standards [20]. Firstly, the desired amount of water and superplasticizer were added to the cement in the mixing bowl and mixed for 3 min. Then, the sand and PP fibers were mixed well, added to the mix, and manually mixed for another 3 min to get the PP fiber-reinforced mortar. The cement mortar was casted in 50 × 50 × 50 mm^3^ cube molds for the compressive strength test. The flexural specimens were casted in 40 mm × 40 mm × 160 mm prisms. The specimens were demolded after 24 h and were cured in lime-saturated water for 28 days. At the end of the curing period, the specimens were extracted from the water, dried, and then heated. 

### 2.3. Heating and Test Procedures 

**Figure 1:**
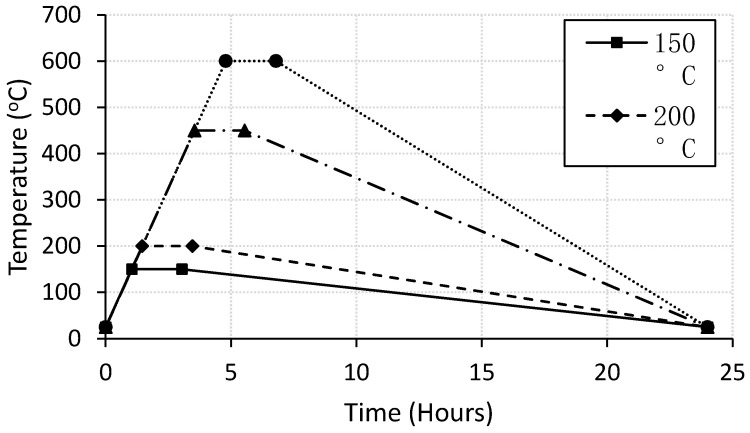
Temperature-time profiles.

The specimens were subjected to the desired elevated temperature using a special electrical furnace with a heating rate of 2 °C/min. The temperature was fixed for two hours at the chosen temperature, then the furnace was turned off, and specimens were left inside it to cool to the room temperature. The temperature-time profiles are shown in Figure 1. The specimens were designated by letters and numbers. The letter “C” was used for control specimens and the letters “PPF” were used for PP fiber-reinforced specimens. The numbers referred to the exposed temperature.

Compressive and flexural strength tests were carried out using a universal testing machine with loading rates of 1.3 kN/s and 2.64 kN/min, respectively. ASTM C109/C109M [21] and ASTM C348-18 [22] standards were followed to perform the compressive and flexural tests, respectively. Three specimens were tested for each mix and the average value was reported. Small fragments were extracted from selected specimens after the compressive test in order to perform the microstructural and thermal analysis. The fragments were obtained from different locations through the specimens to represent the entire sample. SEM imaging was performed according to ASTM C1723-10 [23] on the control and fiber-reinforced mortar exposed to different temperatures in order to monitor the microstructural changes of the specimens due to elevated temperatures. Gold coating was applied on the surface of the fragments to enhance their conductivity. Then, the SEM imaging was conducted using a NOVA NanoSEM 450 device (Hillsboro, Oregon, USA) at high voltage of 5 kV and 20 kV and working distance ranging from 4.6 to 9 mm. The mineralogical and chemical composition analysis were performed through X-ray diffraction (XRD) and X-ray fluorescence (XRF) test procedures. XRD and XRF tests were conducted to examine the chemical composition change of fiber-reinforced mortars due to high temperatures exposure. Small fragments were obtained from selected specimens after the compressive test. The fragments were grinded using a ball-milling machine to pass through a 325-mesh sieve. The powder was placed on the JSX 3201 M (Jeol) (Peabody, MA, USA) spectroscopy machine to conduct the elemental tests. The effect of PP fibers on the thermal behavior of cement mortar was explored using differential scanning calorimetry (DSC) and thermogravimetric analysis (TGA) tests. Mortar specimens were firstly grinded using a ball-milling machine to a size pass through a 325-mesh sieve (45 µm). The specimens were then tested in temperature range from 30 °C to 750 °C, with a heating rate of 5 °C/min. The DSC test was conducted to monitor the changes in the heat capacity of the specimens as changes in the heat flow. The TGA test was performed to identify various phases presented in the mortar specimens and the phase changes due to heating. Thermal conductivity was investigated using a hot disk thermal constant analyzer machine. The transient plane source (TPS) method was used to acquire the thermal conductivity data, as the sensor of the device is sandwiched between two-cement mortars specimens, the mortar specimens used were taken after conducting the flexural strength test. The size of specimens was 40 mm × 40 mm × 80 mm, which was sufficient to cover the full area of the sensor. 

## 3. Results and Discussion

### 3.1. Degradation in Compressive Strength 

**Figure 2:**
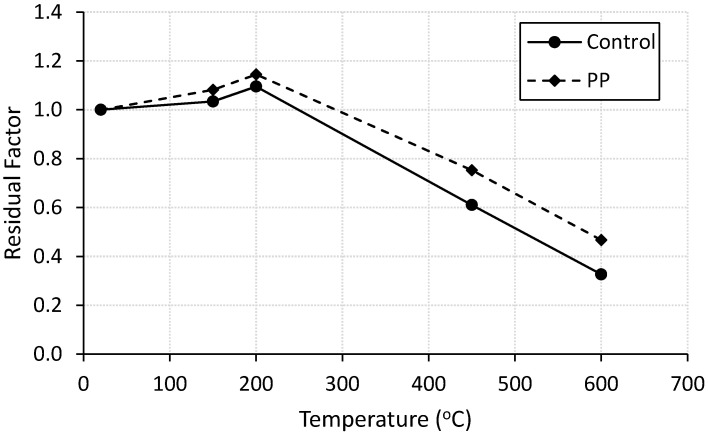
Residual compressive strength factor for control and fiber-reinforced specimens.

Compressive strength results for specimens tested at ambient and elevated temperatures are summarized in Table 3. It is clear that incorporating PP fibers significantly enhances the compressive strength of cement mortar at ambient temperature by 27%. Similar trend was reported in the literatures [24]. The enhancement could be attributed to the role of the PP fibers in bridging the cracks thus resist their propagation and absorb the cracked energy [3,24]. To discuss the effect of PP fibers on the behavior of cement mortar at high temperatures, residual factor for compressive strength of all specimens was calculated as the ratio between strength of exposed specimens and control specimens. Heating specimens up to 200 °C increases the compressive strength of both control and PP fiber reinforced mortar. The enhancement was slightly more visible in the case of PP fiber reinforced specimens as shown in Figure 2. The strength gain up to 200 °C may be due to the degradation of Portlandite [25], or relief of pressures by drying which creates greater van der walls forces thus a closer configuration of capillary pores [16]. Beyond the 200 °C heating cycle, the compressive strength of both control and PP fiber-reinforced mortar reduces due to the degradation of cement when heated specially above 400 °C. However, the influence of PP fiber on the residual compressive strength of mortar mixes is more pronounced as shown in Figure 2. Higher residual factors may be ascribed to the amount of vapour escaping through the microscopic channels formed within the matrix by melting of PP fibers [10,14].

### 3.2. Flexural Behavior Characteristics

**Figure 3:**
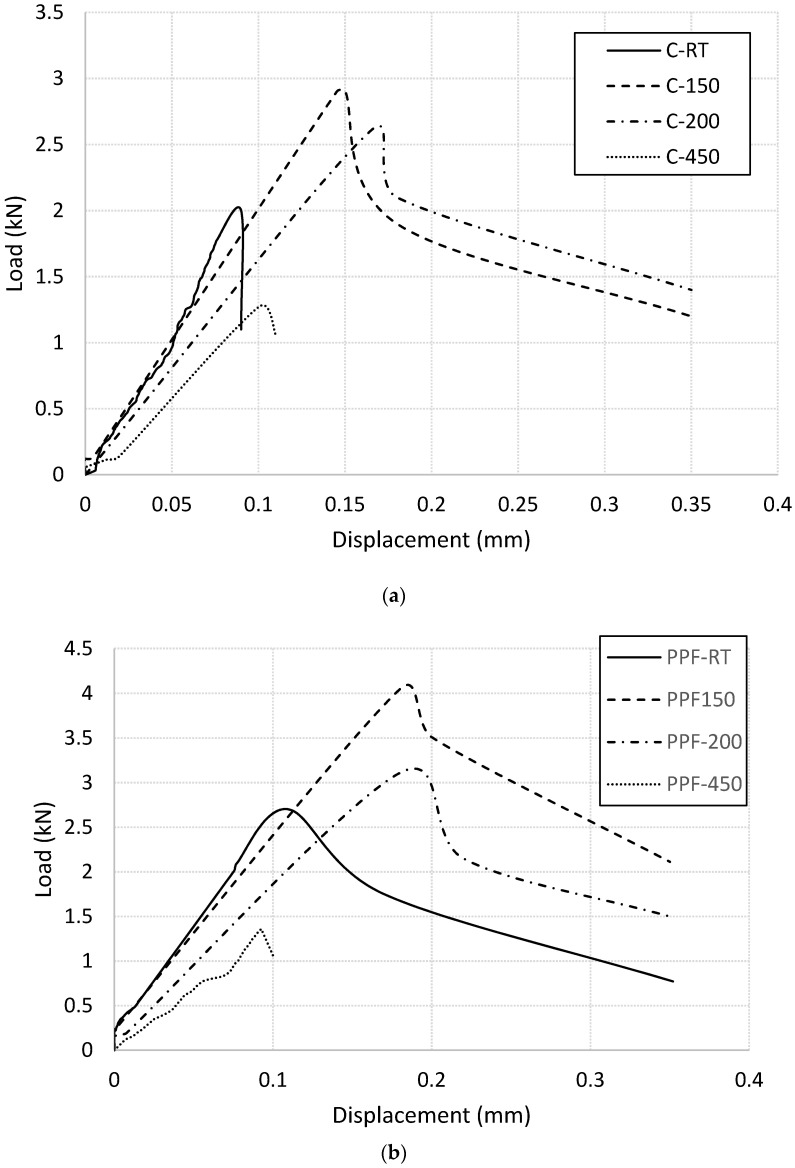
Flexural load-displacement curves at various heating levels (**a**) control specimens (**b**) fiber-reinforced specimens.

The load-displacement curves for control and fiber-reinforced mortar specimens heated at different temperature levels were extracted from flexural test and are plotted in Figure 3. These curves were characterized in terms of flexural strength, modulus of elasticity, and fracture energy. The results were used to calculate the stress intensity factor. Table 3 summarizes the test results. It should be mentioned that the results for flexural specimens are presented until 450 °C heating level. For 600 °C heating level, sever cracking has been occurred in the specimens and hence the results were not reliable and omitted.

#### 3.2.1. Residual Flexural Strength 

**Figure 4:**
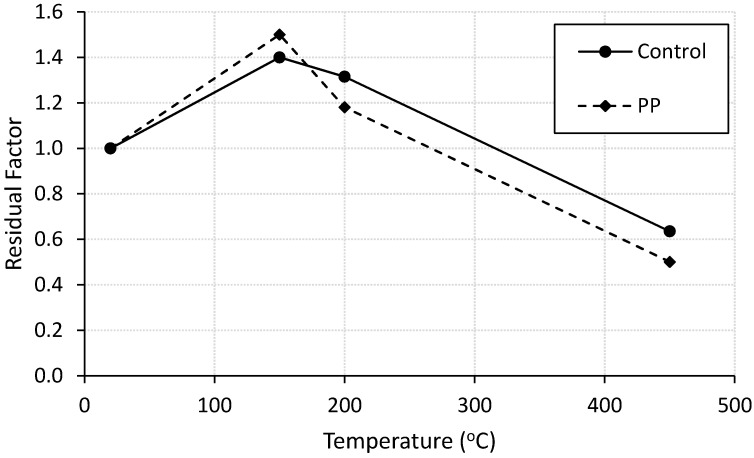
Residual flexural strength factor for control and fiber-reinforced specimens.

Residual flexural strength factor was calculated for plain and fiber-reinforced mortar specimens as the ratio between the ultimate-load of heated specimen divided by the ultimate-load of corresponding control specimen. The results are plotted in Figure 4. Unreinforced mortar specimen showed brittle failure at room temperature. It reached its maximum flexural strength of 2.0 kN then failed directly without any post-failure performance. Incorporating PP fibers into the mortar significantly improved its flexural strength and clearly enhanced its ductility. Heating the mortar up to 200 °C enhanced its residual flexural strength for both unreinforced and fiber-reinforced specimens as shown in Figure 4. After that, the residual flexural strength decreased with increasing the temperature. However, Figure 4 reflects that the polypropylene fibres have a positive role until almost 170 °C, which is close to the melting temperature of the fibers. Beyond this temperature, the fiber-reinforced specimens experience lower residual strength factor than that of unreinforced mortar. 

It was noticed that the residual flexural strength factor of PP fiber-reinforced mortar specimens are higher than the residual compressive strength factor for heating level below 200 °C. This finding reflects the ability of the fibers to reduce the deteriorating effect of elevated temperatures below 200 °C on flexural strength specimens more than that of compressive strength specimens. This behavior may be attributed to the fact that beyond this temperature the fiber melted and could not help in mitigating the destructive effect of micro-cracks that form at higher temperatures due to the tensile stresses created in flexural tests [16,26].

#### 3.2.2. Residual Modulus of Elasticity 

**Figure 5:**
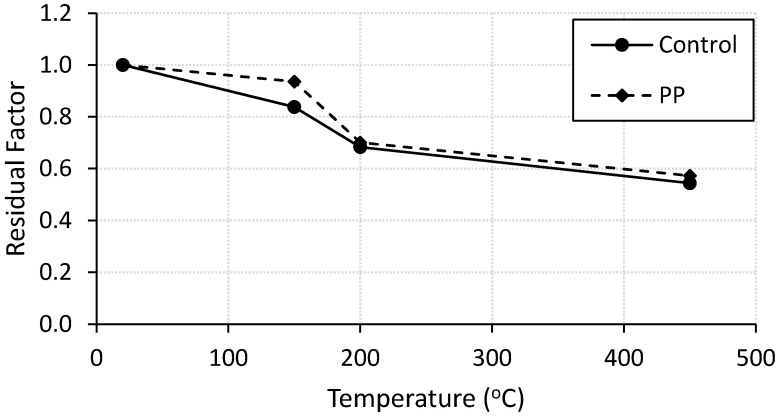
Residual elastic modulus factor for control and fiber-reinforced specimens.

The residual modulus of elasticity of all specimens are calculated as the slope of the linear part of the load-displacement curve and summarized in Table 3. The residual modulus factor was calculated for plain and fiber-reinforced mortar specimens as the ratio between the modulus of heated specimen divided by the modulus of corresponding control specimen. Figure 5 shows the residual modulus factor for all specimens. It was noticed that the residual modulus of elasticity decreases with increasing the heating level. The plain and fiber-reinforced specimens lost almost 45% and 43% of their elastic modulus, respectively, until 450 °C. According to the results, the residual modulus of fiber-reinforced mortar specimens is higher than that of plain mortar specimens for all heated levels. However, the effect of the fibers on the residual elastic modulus is more pronounced at temperatures below 200 °C as shown in Figure 5. similar trend was reported in the literature [13,14,16].

#### 3.2.3. Residual Fracture Energy

**Figure 6:**
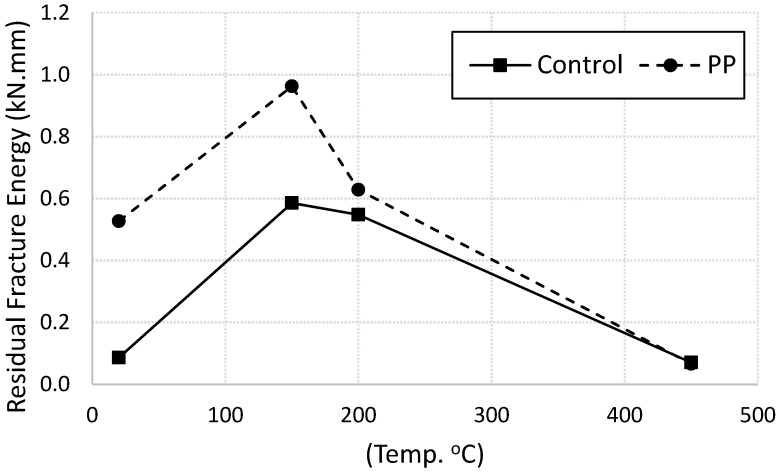
Residual fracture energy for control and fiber-reinforced specimens.

Fracture energy of plain and fiber-reinforced mortar specimens were calculated as the area under the load-displacement curves shown in Figure 3. The values are summarized in Table 3 and plotted in Figure 6. The residual fracture energy of both plain and fiber-reinforced specimens increased with increasing the heating level up to 150 °C. After that, it decreased with increasing the temperature. It is clear that the PP fibers enhanced the residual fracture energy for specimens heated up to 150 °C. Beyond this temperature, the role of the fibers is less pronounced. For specimens heated up to 150 °C, when cracks initiated at the maximum flexural strength, the presence of the PP fibers played a major role to bridge the cracks, distribute the stresses, preventing the crack face separation, and mitigate crack propagation. The fibers continued carrying the load until they pulled out from the mortar. This mechanism offers an extra energy absorption thus higher fracture energy. Similar trend was reported in the literates [4,27,28]. At higher temperatures, the polypropylene fibres start melted thus lost their ability to mitigate the cracks and to stop crack propagation. That leads to sharp drop in the values of fracture energy for fiber-reinforced specimens at heating level between 150 °C and 200 °C (close to the melting temperature of PP fibers) as shown in Figure 6. 

#### 3.2.4. Stress Intensity Factor

Stress intensity factor (KI) is a representative parameter that describes the overall damage behavior of fiber-reinforced mortar. It depends on the Young modulus (E) and fracture energy (Ef) of the specimen as described in Equation (1):

KI = (E × Ef)^0.5^ (1)

**Figure 7:**
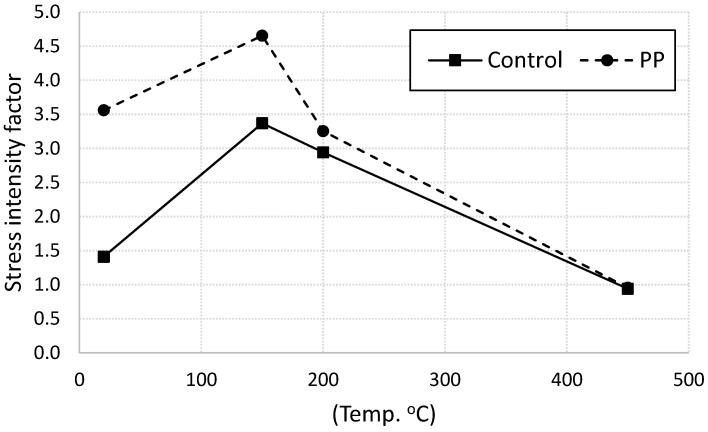
Stress intensity factor for control and fiber-reinforced specimens.

Stress intensity factor of plain and fiber-reinforced mortar specimens subjected to various heating levels were calculated, are listed in Table 3, and are plotted in Figure 7. It is clear that the presence of PP fiber significantly increase the stress intensity factor of cement mortar for heating level below 150 °C. This trend reflect the ability of fibers to delay cracks initiation and mitigate cracks propagation through the mortar specimens. At higher heating level, the difference in the stress intensity factor between plain and fiber-reinforced specimens start decreasing due to fiber melting. A similar trend was reported in [4]. 

#### 3.2.5. Flexural Damage Behavior

**Figure 8:**
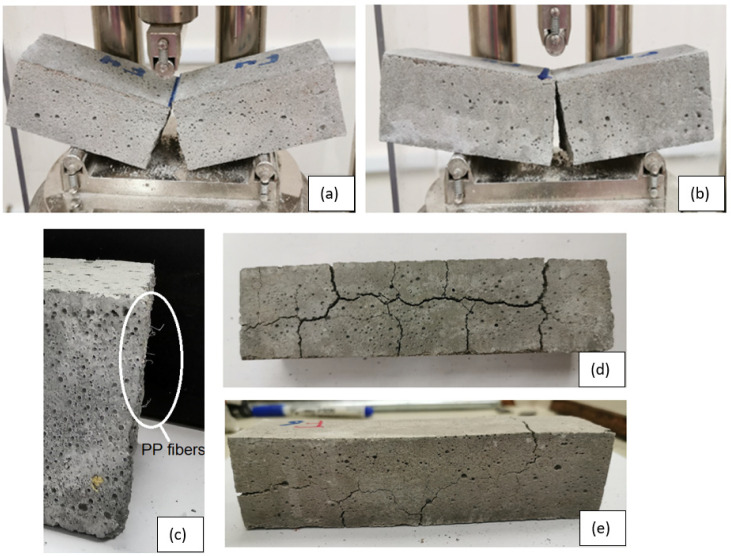
Typical flexural failure mode for: (**a**) plain mortar specimens at room temperature, and heated up to 450 °C; (**b**) fiber-reinforced mortar specimens at RT and heated up to 200 °C; (**c**) polypropylene (PP) fibers at the fracture surface of specimen tested at RT and sever cracks due to heating at 600 °C; (**d**) plain mortar specimen (**e**) fiber reinforced mortar specimen.

Figure 8 shows typical failure modes of plain and fiber-reinforced mortar specimens under flexural loading. At room temperature, plain cement mortar specimen showed brittle failure. It reached its maximum flexural strength then failed directly at low deflection value without any post-failure performance. Incorporating PP fibers into the mortar significantly improved its flexural strength and clearly enhanced its ductility. The PP fibers visually appeared and distributed within the matrix just like micro-reinforcing bars as shown in Figure 8c. These fibers reduce the cracks development and mitigate their propagation thus enhance the ductility of mortar specimens. Heating the plain and fiber-reinforced mortar specimens up to 200 °C did not change their flexural failure mode. However, heating those specimens at 450 °C significantly reduce their ductility, which support the load-deflection diagrams shown in Figure 3. In the case of heating level of 600 °C, plain mortar specimen expertized major and wide cracks at its all faces due to heating as shown in Figure 8d. The flexural test could not carry out due to these cracks thus the results of this specimen was omitted. The presence of PP fibers significantly reduce the intensity and the width of these cracks as shown in Figure 8e. This finding support the hypothesis that melting the PP fibers due to heating create extra pores in the microstructure of concrete thus allow the water steam generated due to the dehydration process to release and hence reduce the spalling.

### 3.3. Thermal Behavior 

#### 3.3.1. Thermal Conductivity

**Figure 9:**
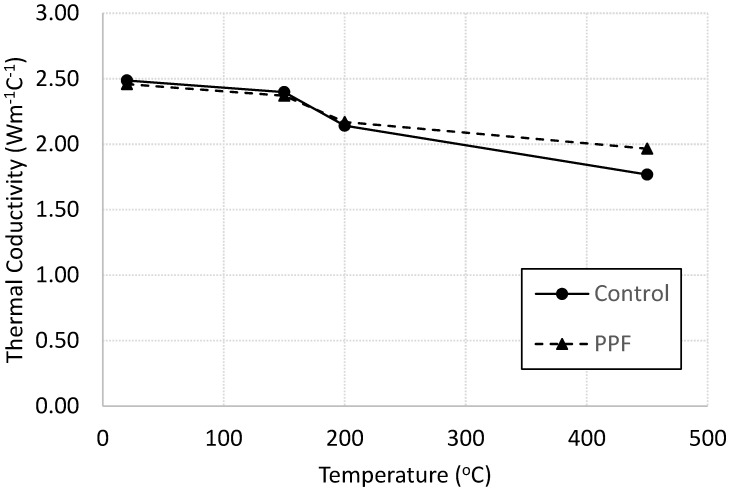
Thermal conductivity of control and fiber-reinforced specimens heated at various levels.

Thermal conductivity of plain and PP fiber-reinforced mortar specimens heated at various temperatures are shown in Figure 9. It is clear that the thermal conductivity of cement mortar decreases with heating regardless the presence of the fibers. Similar trend was reported in [29]. The reduction in thermal conductivity could be attributed to the decomposition of hydration products thus the damage due to heating [29]. Beyond 200 °C, the thermal conductivity of fiber-reinforced mortar is greater than that of plain mortar. This attitude could be linked to the role of the PP fibers in reducing the micro cracks and the damage formulated due to heating. When heated at 450 °C, the polypropylene fibers melted and created extra voids as shown in the SEM images later in this paper. These voids played a major role to allow the steam generated due to the dehydration process to release, thus reduced the internal pore pressure. This internal pressure usually causes severe damage to the microstructure (in the case of plain mortar specimens), and thus may cause a reduction in the thermal conductivity for specimens without fibers. 

#### 3.3.2. Heat Flow Analysis Using Differential Scanning Calorimetry (DSC) 

**Figure 10:**
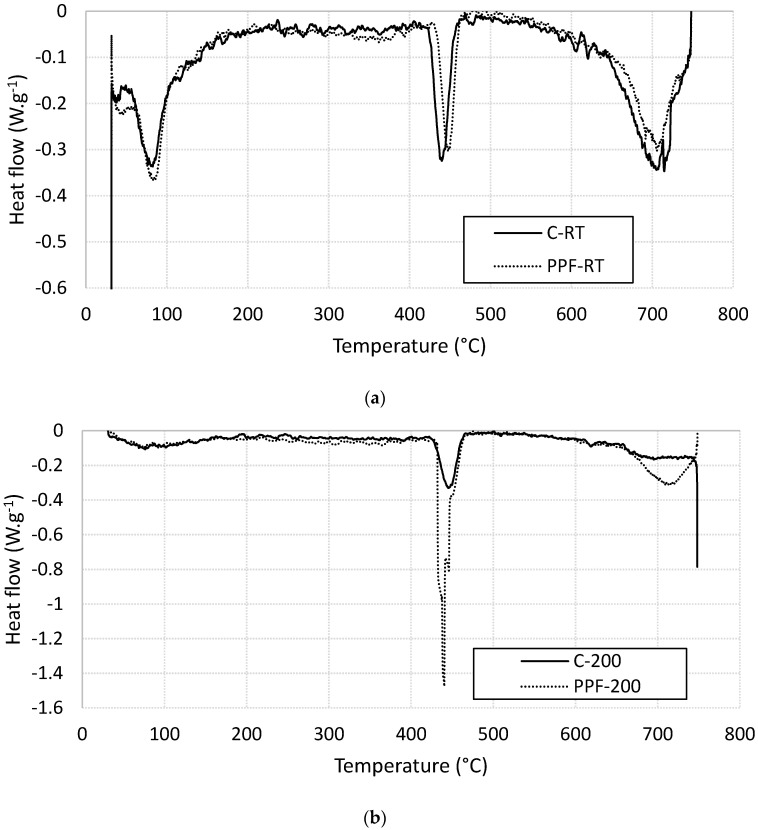
Differential scanning calorimetry (DSC) thermographs for plain and fiber-reinforced specimens (**a**) at room temperatures (**b**) heated at 200 °C.

Heat flow analysis of plain and fiber-reinforced cement mortar specimens were conducted using DSC technique. For unheated specimens, three significant endothermic peaks can be observed within the studied temperature range as shown in Figure 10a. The first peak is located in the interval of 60–120 °C which refers to the decomposition of calcium silicate hydrate (C-S-H) gel [25,30]. The peak temperature of the C-S-H decomposition did not affected by the presence of the PP fibers. The second peak is located in the interval of 420–475 °C which refers to the decomposition of calcium hydroxide (portlandite) [25,30]. The peak temperature of portlandite decomposition is shifted toward higher dissociation temperature in the presence of PP fibers. The third peak is located in the interval 650–750 °C which refers to the decomposition of calcite (CaCO_3_) and carbonated C-S-H [30]. The carbonation of C-S-H leads to the formation of vaterite, which is an unstable type of calcium carbonate. The presence of PP fibers decreases the area of this peak as shown in Figure 10a. For specimens heated at 200 °C, one clear endothermic peak can be observed within the studied temperature range as shown in Figure 10b. This peak is located in the interval of 420–475 °C which refers to the decomposition of calcium hydroxide (portlandite) [25,30]. The portlandite-decomposition peak temperature area significantly increases for specimen contains PP fibers.

#### 3.3.3. Mass Loss Analysis Using Thermogravimetric Analysis (TGA)

**Figure 11:**
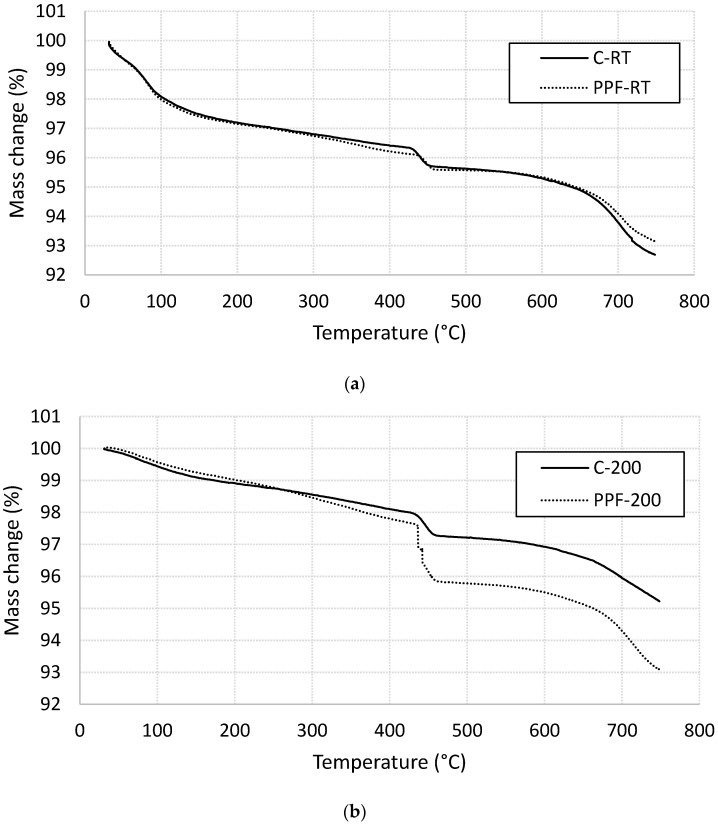
Thermogravimetric profiles for plain and fiber-reinforced specimens (**a**) at room temperatures (**b**) heated at 200 °C.

Mass loss analysis of plain and fiber-reinforced cement mortar specimens were conducted using TGA technique. The Thermogravimetric profile of unheated specimens shown in Figure 11a shows two main mass loss steps. The first one is located at 85 °C related to the decomposition of C-S-H. The mass loss was not affected with the presence of PP fibers, which support the DSC findings. The second mass loss is observed at about 450 °C, which refers to the Portlandite decomposition. The mass loss is not affected by the presence of PP fibers; however, the dissociation temperature is shifted toward higher value. This finding is consistent with the aforementioned DSC results. For specimens heated at 200 °C, one main mass loss step is visible in Figure 11b. This step is located at 450 °C and refer to the decomposition of Portlandite [25,30]. The presence of PP fibers significantly increase the mass loss at this temperature, which is consistence with the increase of the area under the DSC curves. The increase in the mass loss at this stage may be attributed to the melting of the PP fibers due to heating the specimen at 200 °C (greater than melting temperature of PP fibers).

### 3.4. Chemical Composition Using X-ray Diffraction (XRD)

**Figure 12:**
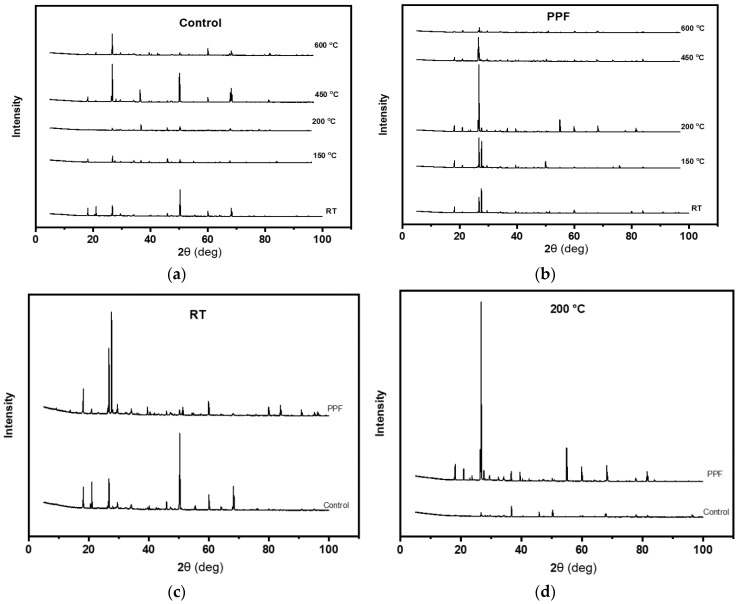
X-ray diffraction (XRD) patterns for (**a**) plain and (**b**) fiber-reinforced specimens heated at various temperatures and XRD patterns comparison (**c**) at room temperature and (**d**) at 200 °C.

Figure 12a,b show the XRD analysis results for plain and fiber-reinforced cement mortar specimens heated at various temperatures. The main phases recognised in all figures are calcium silicate hydrate (CSH) at 2*θ* = 28°, calcium hydroxide (CH) at 2*θ* = 18°, and calcium carbonate (CC) at 2*θ* = 21°. Figure 12b highlights the effect of heating on the XRD patterns of cement mortar with PP fibers. The main finding from this figure is that the intensity of the CSH diffraction at 2*θ* = 28 increased when heating the specimens up to 200 °C then decreased. This finding supports the mechanical strength results. It is also clear that the intensity of the CH diffraction at 2*θ* = 18 is hardly seen for all temperatures. It is obvious in Figure 12c,d that the intensity of the CSH diffraction at 2*θ* = 28 for fiber-reinforced specimens is higher than that of plain mortar specimens tested at room temperature and heated at 200 °C. The reason beyond this trend could be the ability of the polypropylene fibers to accelerate the cement hydration process [31]. These results are similar to those reported in the literature [31]. This finding support the superiority of the residual compressive strength for specimens with PP fibers previously reported in Figure 2. 

### 3.5. Microstructure Investigation Using Scanning Electron Microscopy (SEM)

**Figure 13:**
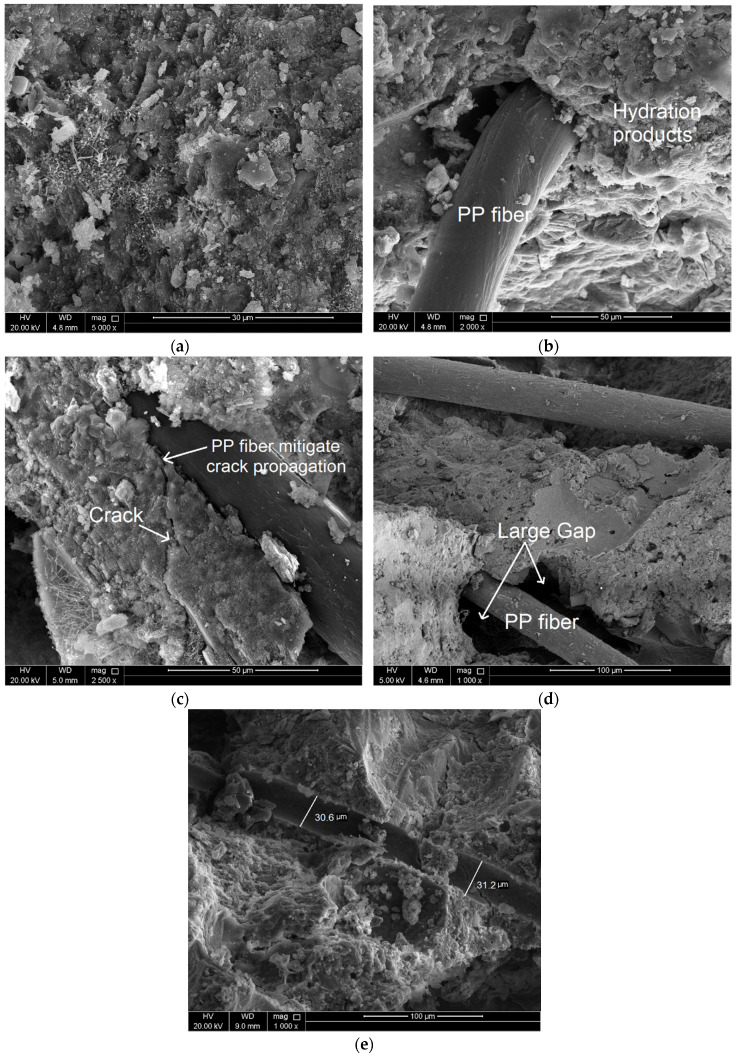
Scanning electron microscope (SEM) images show (**a**) Typical hydration products of cement mortar at room temperature. (**b**) Good bond between PP fiber and mortar debris at room temperature. (**c**) PP fiber bridges the cracks for mortar heated at 150 °C. (**d**) Large gap between PP fiber and mortar debris after testing. (**e**) Empty spaces in the original location of the PP fibers for specimen heated at 450 °C.

SEM analysis was conducted to explore the role of PP fibers in resisting the microstructure deterioration of cement mortar due to heating. At room temperature, typical hydrated products such as calcium silicate hydrates (CSH), calcium hydroxides (CH), and ettringite needles were observed in the micrograph shown in Figure 13a,b shows PP fiber surrounded by mortar debris, which refer to the good bond between the fibers and the hydration products [32]. The bond enhances the stress transfer process thus improve the mechanical strengths [25]. For specimens heated at 150 °C, thermal cracks started to initiate and propagate. Presence of PP fibers plays a major role to bridge the cracks and mitigate their propagation [33] as shown in Figure 13c. However, a large gap was noticed between the fibers and the mortar after testing as shown in Figure 13d. This gap might refer to no chemical bond between the PP fibers and the hydration products was generated [32]. Heating the specimens at 450 °C resulted in fiber melting. Once the melting point of the polypropylene fibers is exceeded, the fibers melt and create extra voids in the microstructure of concrete. Figure 13e shows empty spaces in the original location of the PP fibers for specimen heated at 450 °C. The additional voids allow the steam generated due to the dehydration process to release thus reduce the pore pressure and could delay the spalling [11]. Generating extra voids within the cement matrix represent one of the factors that clearly reduce the mechanical strengths and thermal conductivity of tested specimens as mentioned in previous sections.

## 4. Conclusions

Thermal properties, residual fracture energy, and chemical composition degradation of cementitious composites after being subjected to elevated temperatures are important parameters to estimate the ability of cementitious specimens to survive after the event of fire. The current study investigates the effect of PP fibers on the mechanical and thermal properties of cement mortar after being exposed to elevated temperatures of 150 °C, 200 °C, 450 °C, and 600 °C. The effect of heating level and mixture design on the residual compressive strength, residual flexural strength, modulus of elasticity, failure pattern, fracture energy, stress intensity factor, thermal conductivity, microstructure, and chemical composition degradation is experimentally studied. The following conclusions could be drawn:

1. Adding small dosage of polypropylene (PP) microfibers into cementitious composites enhances their mechanical properties at ambient temperature. Compressive strength, flexural strength, ductility, and elastic modulus of cement mortar are clearly improved by incorporating the PP fibers.
2. Incorporating PP fibers enhances the residual compressive strength of cement mortar when exposed to elevated temperatures. The enhancement is more pronounced for heating level beyond 200 °C.
3. Polypropylene microfiber addition enhances the post-heating flexural behavior of cement mortar. The enhancement is less visible when the heating level exceeds the melting temperature of the fibers (170 °C).
4. Thermal conductivity of cement mortar decreases with heating regardless the presence of the fibers. Beyond 200 °C, the thermal conductivity of fiber-reinforced mortar is greater than that of plain mortar.
5. The presence of PP fibers does not affect the heat flow and the mass loss of cement mortar at room temperature. Heating cement mortar at temperature beyond the melting point of the fibers negatively affect its thermal behavior.
6. The presence of PP fibers plays a major role in bridging the cracks and mitigating their propagation. Once the melting point of the polypropylene fibers is exceeded, the fibers melt and create extra voids in the microstructure of concrete. These extra voids negatively affect the mechanical strengths of the mortar but could enhance spalling resistance.

## Figures and Tables

**Table 1:** Chemical composition of Portland cement.

Compound Name	CaO	SiO_2_	Fe_2_O_3_	SO_3_	Al_2_O_3_	MgO	Na_2_O	LOI
**Content Percentage**	66.4%	18.4%	6.1%	3.0%	2.2%	1.4%	0.8%	1.7%

**Table 2:** Mix design.

Mortar Batch	Cement (Kg/m^3^)	Sand (Kg/m^3^)	Water (Kg/m^3^)	PP Fibers (Kg/m^3^)	Superplasticizer (Kg/m^3^)
1	706.67	1943.33	342.67	-	7.06
2	706.67	1943.33	342.67	0.706	7.06
3	706.67	1943.33	342.67	0.353	7.06

**Table 3:** Mechanical properties of plain and fiber-reinforced specimens exposed to different temperatures.

Specimen	Compressive Strength (MPa)	Flexural Strength (kN)	Elastic Modulus (Gpa)	Fracture Energy (kN.mm)	Stress Intensity Factor
C-RT	30.3 (29.2–31.4)	2.00 (1.88–2.12)	23.134	0.086	1.41
C-150	31.3 (30.4–32.2)	2.80 (2.7–2.9)	19.38	0.586	3.37
C-200	33.2 (32.0–34.4)	2.63 (2.52–2.74)	15.8	0.548	2.94
C-450	18.5 (16.8–20.2)	1.27 (1.07–1.47)	12.58	0.070	0.94
C-600	9.9 (8.6–11.2)	0.21 (0.06–0.36)	NA	NA	NA
PPF-RT	38.5 (37.5–39.5)	2.70 (2.59–2.81)	24.04	0.527	3.56
PPF-150	41.7 (40.4–43.0)	4.05 (3.86–4.24)	22.5	0.963	4.65
PPF-200	44.1 (42.6–45.6)	3.19 (3.04–3.34)	16.85	0.628	3.25
PPF-450	29.0 (27.6–30.4)	1.35 (1.23–1.47)	13.77	0.066	0.96
PPF-600	18.0 (16.2–19.8)	NA	NA	NA	NA

Numbers in parentheses are the range in MPa.
